# Proteomic and Metabolomic Analyses of Lung Squamous Cell Carcinoma

**DOI:** 10.3390/biomedicines14102389

**Published:** 2026-10-19

**Authors:** Yijun Mo, Lina Lin, Jianhua Zhang, Qing Li, Daochuan Li

**Affiliations:** 1 Cardiothoracic Surgery, Shenzhen Hospital, Southern Medical University, Shenzhen 518100, China; 13711239326@163.com (Y.M.); zhangjianhua@smu.edu.cn (J.Z.); 2 School of Nursing, Guangzhou Xinhua University, Guangzhou 510520, China; linlina1764@163.com; 3 Institute of Toxicology, Guangdong Provincial Center for Disease Control and Prevention, Guangzhou 511430, China; 4 Department of Toxicology, School of Public Health, Sun Yat-sen University, Guangzhou 510080, China

**Keywords:** lung squamous cell carcinoma, proteomics, metabolomics, cell adhesion, amino acid metabolism, pilot study

## Abstract

**Background/Objectives**: As the leading cause of global cancer mortality, lung cancer suffers from low survival rates and an incomplete understanding of its pathogenesis. Lung squamous cell carcinoma (LSCC) involves dynamic protein and metabolic dysregulation. To gain initial molecular insights into LSCC is therefore essential to explore coordinated molecular alterations and early mechanisms in primary LSCC tissues. **Methods**: In this pilot study, we integrated proteomic and non-targeted metabolomic profiling to analyze six paired human LSCC tissue specimens and matched adjacent non-cancerous tissues. **Results**: Preliminary analyses revealed extensive differences at both the proteomic and metabolomic levels. Specifically, proteomic profiling highlighted widespread dysregulation in cell adhesion dynamics, whereas non-targeted metabolomic profiling identified perturbations in several amino acid metabolic pathways. Integrated multi-omics analysis suggested potential co-variation patterns linking endoplasmic reticulum (ER) protein processing, cell adhesion, and amino acid metabolism. **Conclusions**: Collectively, these pilot findings provide an initial exploratory characterization of paired proteomic and non-targeted metabolomic alterations in LSCC. Given the small cohort size, these results serve as hypothesis-generating molecular signatures, and all findings require confirmation in larger, independent cohorts with orthogonal and functional validation.

## 1. Introduction

Lung cancer represents a formidable global public health challenge and remains the leading cause of cancer-related death worldwide [1]. Approximately 2.6 million new lung cancer cases and 1.8 million lung cancer deaths were estimated globally in 2024, and the burden continues to increase in many regions [1]. Despite recent advances in treatment, the 5-year net survival rate remains between 10% and 20%, among the lowest across all malignancy types [2]. Lung squamous cell carcinoma (LSCC) accounts for approximately 25–30% of all non-small cell lung cancer (NSCLC) cases [3,4]. Unlike lung adenocarcinoma, LSCC lacks classic actionable driver mutations and is typically characterized by abundant stroma and intense immune evasion [5,6]. Consequently, dissecting the intrinsic molecular characteristics of LSCC is imperative for enhancing diagnostic sensitivity and clinical therapeutic efficacy.

Numerous proteomic studies have illuminated the complex molecular landscape of LSCC [7,8,9]. As proteins serve as the primary functional executioners within cells, proteomic profiling provides critical molecular insights and a clinical translational foundation for identifying potential diagnostic and prognostic biomarkers in LSCC [10]. However, integrating proteomic data with other omics modalities can foster a far deeper understanding of intra-tumoral processes [10,11]. Metabolomics captures rich information regarding dynamic metabolic alterations, serving as the ultimate functional readout of biological activity. Building upon this, Momcilovic et al. demonstrated that LSCC harbors a highly conserved metabolic phenotype that can be therapeutically targeted independently of underlying genetic driver mutations [12]. The synergy of integrated proteomics and metabolomics offers a more holistic vantage point than either approach alone, holding promise for advancing the understanding of LSCC oncogenesis and progression.

In this pilot study, we integrated data-independent acquisition (DIA) proteomic and non-targeted metabolomic profiling to explore the molecular alterations that distinguish LSCC tissues from paired adjacent non-cancerous tissues, with the aim of generating hypotheses about candidate functional proteins and metabolic reprogramming events. Our exploratory findings suggest that altered cell adhesion states and aberrant amino acid metabolism are associated with LSCC, and integrated multi-omics analysis indicates a possible coordinated interplay. These observations provide valuable evidence for deciphering the pathogenic network of LSCC and informing the design of larger validation studies.

## 2. Materials and Methods

### 2.1. Tissue Samples

For proteomic and metabolomic profiling, six pairs of primary LSCC tissues and matched adjacent non-cancerous tissues were derived from residual diagnostic specimens at Shenzhen Hospital of Southern Medical University. All enrolled patients had a newly confirmed primary diagnosis of LSCC, and individuals with underlying secondary malignancies or confounding pulmonary diseases were excluded. Adjacent non-cancerous tissue was defined and sampled at a distance greater than 2 cm from the macroscopic tumor margin, consistent with published articles for lung tissue omics [13]. Prior to multi-omics extractions, frozen sections of all specimens underwent rigorous independent pathological evaluation via hematoxylin and eosin (H&E) staining. H&E diagnosis confirmed that the tumor tissues exhibited characteristic squamous cell carcinoma morphology—specifically, malignant cells growing in nests and sheets with invasive patterns. Detailed H&E pathological images for all specimens are provided in Supplementary Figure S1. Following surgical collection, tissue specimens were immediately snap-frozen and stored in liquid nitrogen until subsequent multi-omics analysis. The detailed clinical characteristics of the patient cohort are summarized in Table 1.

### 2.2. Data-Independent Acquisition (DIA) Proteomic Profiling

Proteomic analysis was performed by Shanghai BIOTREE Biotech Co., Ltd. (Shanghai, China). Tissue specimens were lysed in RIPA lysis buffer, followed by mechanical homogenization and sonication. The homogenates were centrifuged at 12,000 rpm for 10 min at 4 °C to harvest the supernatant. Protein concentrations were determined using a bicinchoninic acid (BCA) protein assay kit. Following enzymatic digestion and peptide desalting, each sample was analyzed with a nano-UPLC (Vanquish neo) coupled to an Astral Zoom instrument (Thermo Scientific, Waltham, MA, USA) with a nano-electrospray ion source. Separation was performed using a reversed-phase column (EASY-Spray™ HPLC (150 µm × 15 cm, Thermo Scientific). Mobile phases were H_2_O with 0.1% FA (phase A) and 80% ACN with 0.1% FA (phase B). DIA was performed with positive ion detection. The parameters were set as follows: electrospray voltage at 1800 V, temperature at 280 °C; MS1 scan range of 380–980 m/z with a resolution of 240 k, AGC target of 500%, and maximum injection time of 3 ms; MS2 scan range of 150–2000 m/z with a maximum injection time of 3 ms, isolation window of 2 Th, RF-lens at 40%, MS2 activation type of HCD, normalized collision energy of 25%, and a cycle time of 0.6 s. Raw mass spectrometry data were processed using DIA-NN (v2.2.0) in library-free (direct DIA) mode. Precursor and protein identifications were filtered at a false discovery rate (FDR) below 5% (q < 0.05), and protein groups were assembled with the default neural-network scoring of DIA-NN. Four statistical metrics—precursor ion identifications, peptide identifications, protein identifications, and protein group identifications—were evaluated, with protein group counts utilized as the standard qualitative metric for proteome coverage. Protein quantification was derived from the DIA-NN protein-group matrix, in which the abundance of each protein group in a given sample is the summed intensity of its identified precursors. Missing values, which arise mainly from peptides whose abundance is close to the limit of detection, were imputed with half of the minimum value of the quantitative data matrix; the imputed matrix was then log_2_(1 + abundance)-transformed and normalized across samples, and sample-wise abundance distributions and Pearson correlation coefficients were inspected to confirm within- and between-group comparability before differential analysis. Sample preparation order and liquid chromatography-tandem mass spectrometry (LC-MS/MS) injection order were fully randomized, and a pooled quality control (QC) sample was analyzed at regular intervals to monitor instrument performance. Proteins adhering to the selection criteria of |log_2_FC| ≥ 1 and Benjamini–Hochberg FDR-adjusted *p* < 0.05 were considered differentially expressed proteins (DEPs), and only these FDR-controlled DEPs were used for all downstream analyses.

### 2.3. Protein–Protein Interaction (PPI) Network Analysis

To explore functional interactions among the identified DEPs, a protein–protein interaction (PPI) network was constructed using the Search Tool for the Retrieval of Interacting Genes/Proteins (STRING) database (https://string-db.org/). The organism was specified as Homo sapiens, and significant interactions were filtered using an interaction score threshold corresponding to medium confidence, with disconnected nodes hidden from the network layout. Subsequently, the network was rendered and visualized using Cytoscape software (v3.10.5). To mitigate blood-borne contamination common in tissue proteomics, circulating high-abundance proteins—including serum albumin (ALB) and hemoglobin subunits (HBA1 and HBB)—were systematically flagged and removed from the candidate DEP list [14]. Key hub proteins within the contaminant-filtered network were then identified via the cytoHubba plugin based on topological algorithms, primarily Maximal Clique Centrality (MCC) and Degree.

### 2.4. Non-Targeted Metabolomic Profiling

Non-targeted metabolomic analysis was performed by Shanghai BIOTREE Biotech Co., Ltd. (Shanghai, China) using the same cohort of tissue specimens as employed in the proteomic profiling. The LSCC tissue samples (25 mg ± 1 mg) were taken, mixed with beads and 500 μL of extraction solution (MeOH: CAN: H2O, 2:2:1 (v/v)). The extraction solution contained deuterated internal standards. The mixed solution was vortexed for 30 s. Then the mixed samples were homogenized (35 Hz, 4 min) and sonicated for 5 min in a 4 °C water bath; the step was repeated three times. The samples were incubated for 1 h at −40 °C to precipitate proteins. Then the samples were centrifuged at 12,000 rpm for 15 min at 4 °C. The supernatant was transferred to a fresh glass vial for analysis. The quality control (QC) sample was prepared by mixing equal aliquots of the supernatant from all 12 specimens; three QC injections were analyzed together with the experimental samples, and blank samples were interspersed throughout the run to monitor carry-over. Hydrophilic interaction liquid chromatography (HILIC) and reversed-phase liquid chromatography (RPLC) separations were conducted on a Vanquish Ultra-High Performance Liquid Chromatography (UHPLC) system (Thermo Scientific). For HILIC separation, target analytes were resolved using a Waters (Milford, MA, USA) ACQUITY UPLC BEH Amide column (2.1 mm × 50 mm, 1.7 μm). The aqueous mobile phase A comprised 25 mmol/L ammonium acetate and 25 mmol/L ammonium hydroxide in water, while mobile phase B consisted of acetonitrile. For reversed-phase separation, chromatographic resolution was achieved using a Phenomenex Kinetex C18 column (2.1 mm × 50 mm, 2.6 μm) (Phenomenex, Torrance, CA, USA). Mobile phase A was composed of 0.01% acetic acid in water, and mobile phase B comprised isopropanol and acetonitrile (1:1, v/v). For both chromatographic modes, the autosampler temperature was maintained at 4 °C, and the injection volume was set to 2 μL. Raw LC-MS data files were converted to mzXML format using ProteoWizard software (v 3.0.26272-10aac2d) and subsequently processed for metabolite identification via an in-house-developed R package interfaced with the BiotreeDB database. Metabolite identification followed the Metabolomics Standards Initiative (MSI) framework: the measured retention time (RT), precursor m/z (MS1), and MS2 spectra were matched against the in-house BiotreeDB (V4.0) and public spectral libraries (including HMDB, METLIN, and mzCloud). Identifications were annotated as MSI level 1 when MS1, MS2, and RT all matched an authentic reference standard (*n* = 1331), as level 2 when both MS1 and MS2 matched a library entry (*n* = 2685), and as level 3 when the match was based on theoretical spectra with predicted RT (*n* = 223); features supported only by precursor mass or that remained unidentified were recorded as level 4 and were excluded from downstream analysis. Deviations between measured and theoretical precursor m/z values were within 15 ppm for the retained identifications (median 2.5 ppm among level-1 matches), and MS2 matching scores ranged from 1.49 to 3.99 (median 2.64). Across the three QC injections, the median relative standard deviation (RSD) of the 73,803 detected features was 3.2%, and 97.2% of the features showed an RSD below 20%; the internal standard RSDs ranged from 0.19% to 1.94%, and pairwise Pearson correlations between QC samples were 0.9984–0.9995. Retention-time drift was monitored using a mixture of authentic standards (RTQC) injected repeatedly over 24 h; the RT deviation remained below 30 s, and a retention-time correction model was applied (R^2^ > 0.9, slope 0.8–1.2). Data preprocessing consisted of (i) removal of features with an RSD above 30% or a detection rate below 50%, (ii) exclusion of features with more than 50% missing values within a group, (iii) imputation of the remaining missing values with one-half of the minimum value, and (iv) normalization to the total ion current (TIC) of each sample. In total, 73,797 features were retained, of which 4239 metabolites (MSI levels 1–3) were used for the downstream differential analysis. Sample preparation and injection order were randomized. Metabolites meeting the criteria of Variable Importance in Projection (VIP > 1) derived from orthogonal partial least squares–discriminant analysis (OPLS-DA) and unadjusted nominal *p* < 0.05 were considered differentially expressed metabolites (DEMs), and only these DEMs were used for all downstream analyses.

### 2.5. Integrative O2PLS Analysis of Proteomic and Metabolomic Data

To systematically integrate proteomic and metabolomic profiles, two-way orthogonal partial least squares (O2PLS) analysis was performed. O2PLS is a robust multivariate data integration algorithm that decomposes total variance across two datasets into joint predictive components, dataset-specific orthogonal components, and residual noise, thereby uncovering inter-omics correlations while accounting for dataset-specific structural variation. O2PLS modeling was conducted using the OmicsPLS package in R studio (v4.4.1). Prior to analysis, the abundance matrices of differentially expressed proteins (DEPs) and differentially expressed metabolites (DEMs) were log_2_-transformed and Z-score normalized. The optimal number of joint components, proteomics-orthogonal components, and metabolomics-orthogonal components was determined via k-fold cross-validation. Model robustness was additionally assessed by 1000-times permutation testing, and the resulting R2X, R2Y, and Q2 values together with the permutation statistics are reported in Supplementary Figure S2. Loading vectors derived from the joint components were extracted to quantify the strength of association between candidate proteins and metabolites. Subsequently, Spearman correlation analysis paired with hierarchical clustering was applied to explore co-clustering patterns across biological pathways.

### 2.6. Western Blot Analysis

Total protein was extracted using RIPA lysis buffer with protease/phosphatase inhibitors (Beyotime, Shanghai, China) and quantified by a BCA assay (Beyotime). Equal protein amounts (20–30 μg) were separated by SDS-PAGE and transferred onto PVDF membranes (Millipore, Billerica, MA, USA). After blocking with 5% BSA in TBST for 2–4 h, membranes were incubated overnight at 4 °C with primary antibodies against ITGB1 (Proteintech, Wuhan, China, 12594-1-AP), CAV1 (Proteintech, 16447-1-AP), CTNNB1 (Proteintech, 51067-2-AP), CDK1 (Proteintech, 19532-1-AP), TP53 (Proteintech, 60283-2-Ig), and β-actin (Proteintech, 66009-1-Ig) as an internal control. Membranes were washed with TBST, incubated with HRP-conjugated secondary antibodies (1 h, room temperature), and visualized using ECL reagents (Abbkine, Wuhan, China). Band densitometry was analyzed using ImageJ (v1.54) and normalized to β-actin.

### 2.7. Statistical Analysis

For differential expression analyses of proteomic and non-targeted metabolomic datasets, all statistical computations were performed within R Studio. Differential expression profiling was executed using the limma package with a paired design. The intra-patient pairing controls for fixed individual-level covariates such as age, sex, and smoking history. Pairwise comparisons between paired tumor and adjacent non-cancerous tissues were additionally evaluated using paired Student’s t-tests for descriptive purposes. Raw *p* values were adjusted for multiple hypothesis testing using the Benjamini–Hochberg procedure to control the false discovery rate (FDR), and features with an adjusted *p* value (*p*_adj_) < 0.05 were considered statistically significant. The criteria were applied to proteomic (|log_2_FC| ≥ 1 with *p*_adj_ < 0.05), whereas differentially expressed metabolites (DEMs) were identified based on VIP > 1.0 and unadjusted nominal *p* < 0.05 at the discovery stage. Gene Ontology (GO) annotation and Kyoto Encyclopedia of Genes and Genomes (KEGG) pathway enrichment analyses were conducted using Fisher’s exact test, and the resulting pathway-level *p* values were also adjusted with the Benjamini–Hochberg method, with FDR-adjusted *p* < 0.05 defined as statistically significant. The directionality of each enriched metabolic pathway was summarized using a pathway activation Z-score calculated from the log_2_FC of its constituent metabolites, in which positive and negative values indicate prevailing up- or down-regulation, respectively. All enrichment, network, and integration analyses were re-run using only the DEP and DEM lists.

## 3. Results

### 3.1. Proteomic Alterations in Lung Squamous Cell Carcinoma Tissues

**Figure 1:**
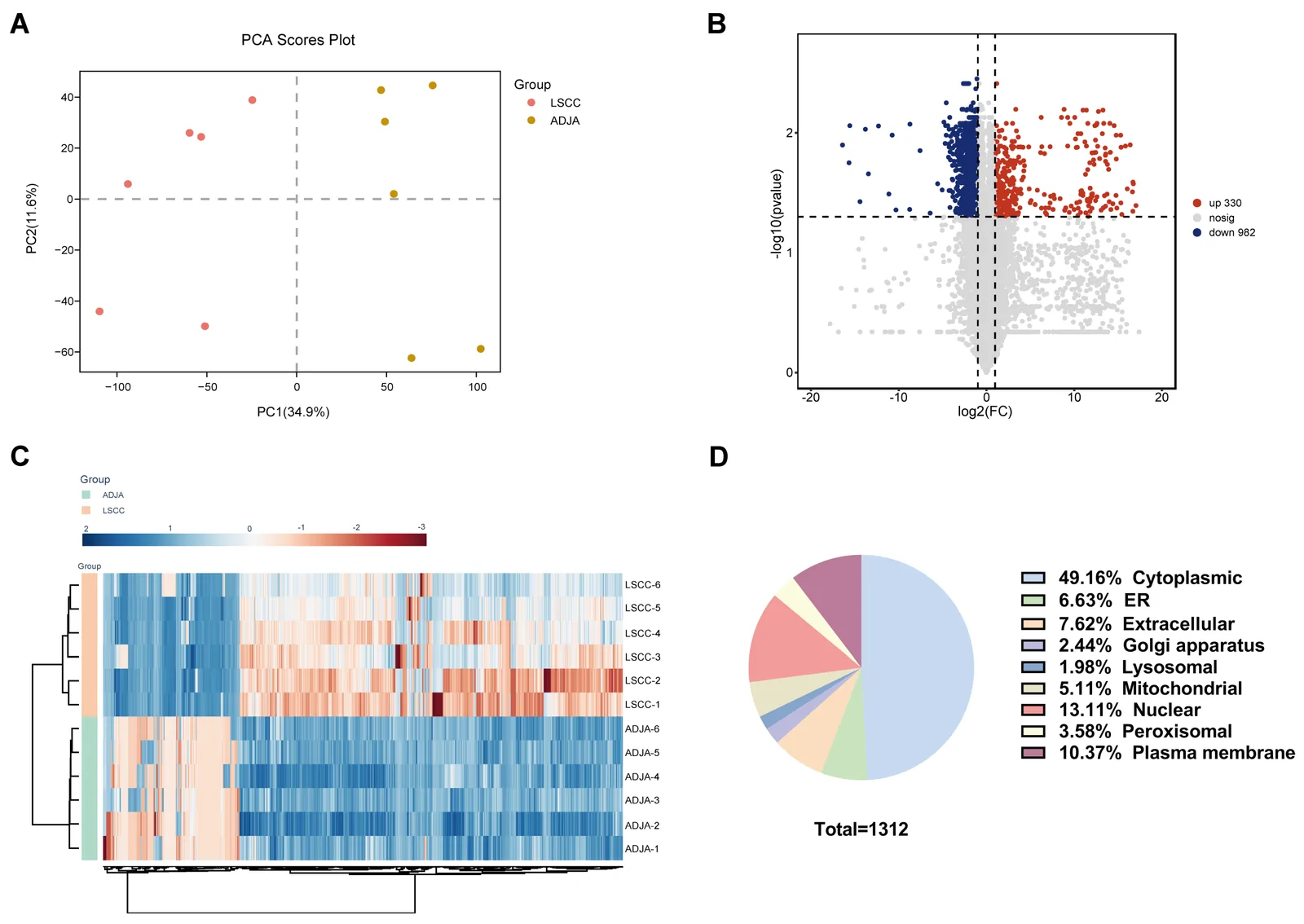
Proteomic characterization of LSCC and adjacent non-tumor tissues. (**A**) PCA score plot based on proteomic profiles of LSCC (*n* = 6) and paired adjacent tissues (ADJA, *n* = 6). (**B**) Volcano plot showing differentially expressed proteins (DEPs) between LSCC and ADJA tissues (red: upregulated, blue: downregulated, gray: non-significant). (**C**) Hierarchical clustering heatmap of total protein expression patterns across samples. (**D**) Subcellular localization and percentage distribution of the 1312 identified DEPs.

Proteomic analysis was conducted on six pairs of lung squamous cell carcinoma (LSCC) and matched adjacent non-tumor tissue (ADJA) samples to identify differences in protein expression between the two groups. Principal component analysis (PCA) revealed that the normalized samples tended to separate according to group on the PCA score plot (Figure 1A). Given that unsupervised multivariate separation is readily achieved in small-cohort datasets, this clustering pattern was not treated as definitive proof of population-level biological divergence. In total, 163,657 peptides and 13,942 protein groups were identified, with 13,011 shared across both groups. Following ID conversion and gene annotation, 10,930 proteins were successfully mapped to official gene symbols, providing a foundation for downstream bioinformatic investigations, including functional annotation and enrichment analysis. Specifically, 1312 proteins met the FDR-controlled significance criteria in LSCC tissues (|log_2_FC| ≥ 1 & adjusted *p* < 0.05), comprising 330 upregulated and 982 downregulated proteins. The volcano plot visually illustrated the overall differential expression profile between LSCC and adjacent tissues (Figure 1B). A hierarchical clustering heatmap further demonstrated distinct proteomic expression patterns between the two groups (Figure 1C). Given the crucial role of subcellular localization in elucidating protein structure, function, and protein–protein interactions, we further characterized the subcellular distribution of these differentially expressed proteins (DEPs). The DEPs were predominantly localized to the cytoplasm, nucleus, and plasma membrane (Figure 1D). Collectively, these exploratory findings suggest that LSCC tissues display a distinct proteomic profile relative to adjacent non-cancerous tissues, which requires confirmation in larger cohorts.

### 3.2. Functional Enrichment Analysis and Protein–Protein Interaction Network of DEPs

**Figure 2:**
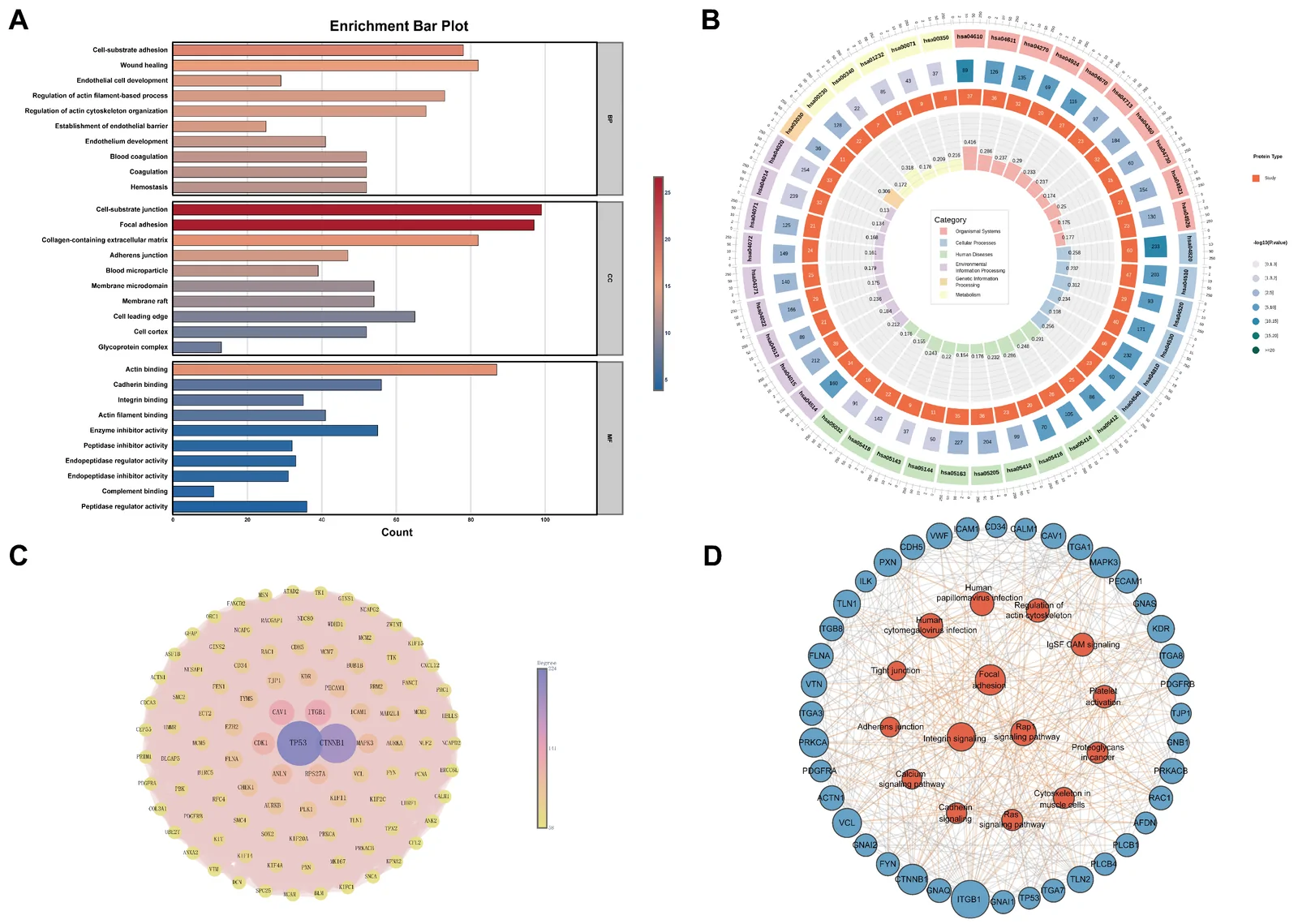
Functional enrichment and protein–protein interaction (PPI) network analyses of DEPs. (**A**) Top 10 significantly enriched Gene Ontology (GO) terms across Biological Process (BP), Cellular Component (CC), and Molecular Function (MF) categories. (**B**) Circos plot showing enriched KEGG pathways categorized by biological system and function. (**C**) PPI network of DEPs with node size and color scaled by node degree. (**D**) Sub-network showing key functional modules and enriched pathway–gene interactions.

To further elucidate the biological roles of the DEPs, Gene Ontology (GO) enrichment analysis was performed across the Biological Process (BP), Cellular Component (CC), and Molecular Function (MF) categories. Figure 2A displays the top 10 significantly enriched terms within the BP, CC, and MF categories. BP terms were primarily associated with cell–substrate adhesion, wound healing, and endothelial cell development; CC terms indicated a predominant distribution in the cell–substrate junction, focal adhesion, and collagen-containing extracellular matrix; and MF classification identified actin binding, cadherin binding, and integrin binding as the three most represented categories by protein count. KEGG pathway enrichment analysis revealed that DEPs were significantly enriched in multiple key pathways (Figure 2B), mainly including cell adhesion-related pathways (focal adhesion, hsa04510; tight junction, hsa04530; adherens junction, hsa04520; and gap junction, hsa04540), cytoskeletal regulation pathways (regulation of actin cytoskeleton, hsa04810; and vascular smooth muscle contraction, hsa04270), immune and vascular response pathways (complement and coagulation cascades, hsa04610; and platelet activation, hsa04611), as well as the oxytocin signaling pathway (hsa04921). Most of these pathways are involved in cell–cell junctions, cell migration, signal transduction, and immune regulation, suggesting that the identified DEPs may mediate phenotypic differences between LSCC and adjacent non-cancerous tissues by modulating cell adhesion, cytoskeletal remodeling, and immune-related signaling cascades.

Investigating protein–protein interactions (PPI) is pivotal for elucidating the functional mechanisms of DEPs. Therefore, we integrated PPI data retrieved from the STRING database with pathway-protein relationships to construct a PPI network and a pathway–gene functional network from the FDR-controlled DEP list. After contaminant proteins (ALB, HBA1, HBB, and other abundant plasma proteins) were removed, the reconstructed PPI network revealed a highly interconnected module centered on hub proteins including TP53, CTNNB1, ITGB1 and so on (Figure 2C). Furthermore, the pathway–gene interaction network underscored the intricate regulatory crosstalk between pivotal enriched pathways—such as focal adhesion, tight junction, and regulation of actin cytoskeleton—and their corresponding key protein components (Figure 2D).

### 3.3. Metabolomic Alterations in Lung Squamous Cell Carcinoma Tissues

**Figure 3:**
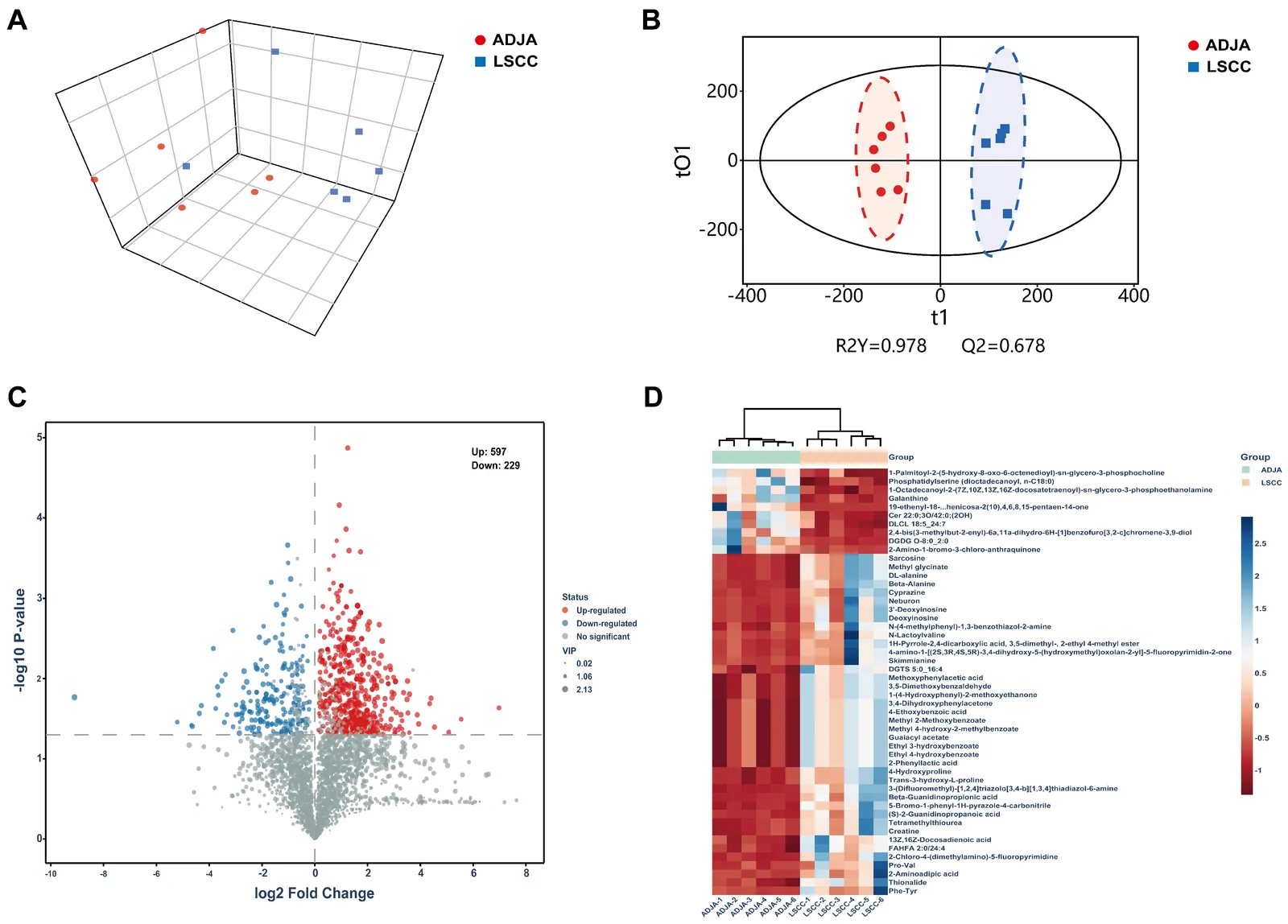
Metabolomic profiling of LSCC and adjacent non-tumor tissues. (**A**) PCA score plot based on metabolomic data of LSCC (*n* = 6) and paired adjacent tissues (ADJA, *n* = 6). (**B**) OPLS-DA score plot displaying separation between LSCC (blue circles) and ADJA (red circles) groups (R^2^Y = 0.978, Q^2^ = 0.678). (**C**) Volcano plot showing DEMs (red: upregulated, blue: downregulated, gray: non-significant). (**D**) Hierarchical clustering heatmap of the top 50 significant DEMs across samples.

PCA showed that the normalized samples tended to separate according to group on the PCA score plot (Figure 3A). Orthogonal partial least squares-discriminant analysis (OPLS-DA) separated the two groups with R2Y and Q2 values of 0.978 and 0.678, respectively (Figure 3B), and a 1000-iteration permutation test yielded an intercept Q2 of −0.54. These separation patterns were treated as preliminary descriptive features, warranting further validation in larger prospective patient cohorts. In total, 73,797 metabolic features and 4239 distinct metabolites were identified. Differentially expressed metabolites (DEMs) were determined using the criteria of Variable Importance in Projection (VIP > 1.0) and *p* < 0.05. The volcano plot illustrates the overall DEM distribution between the two groups (Figure 3C), revealing 597 upregulated and 229 downregulated metabolites. The top 50 differential metabolites ranked by importance are displayed in Figure 3D. Collectively, these exploratory findings suggest that LSCC tissues display a distinct metabolomic profile relative to adjacent non-cancerous tissues, pending validation in larger cohorts.

### 3.4. Functional Annotation and Pathway Analysis of Differentially Expressed Metabolites

**Figure 4:**
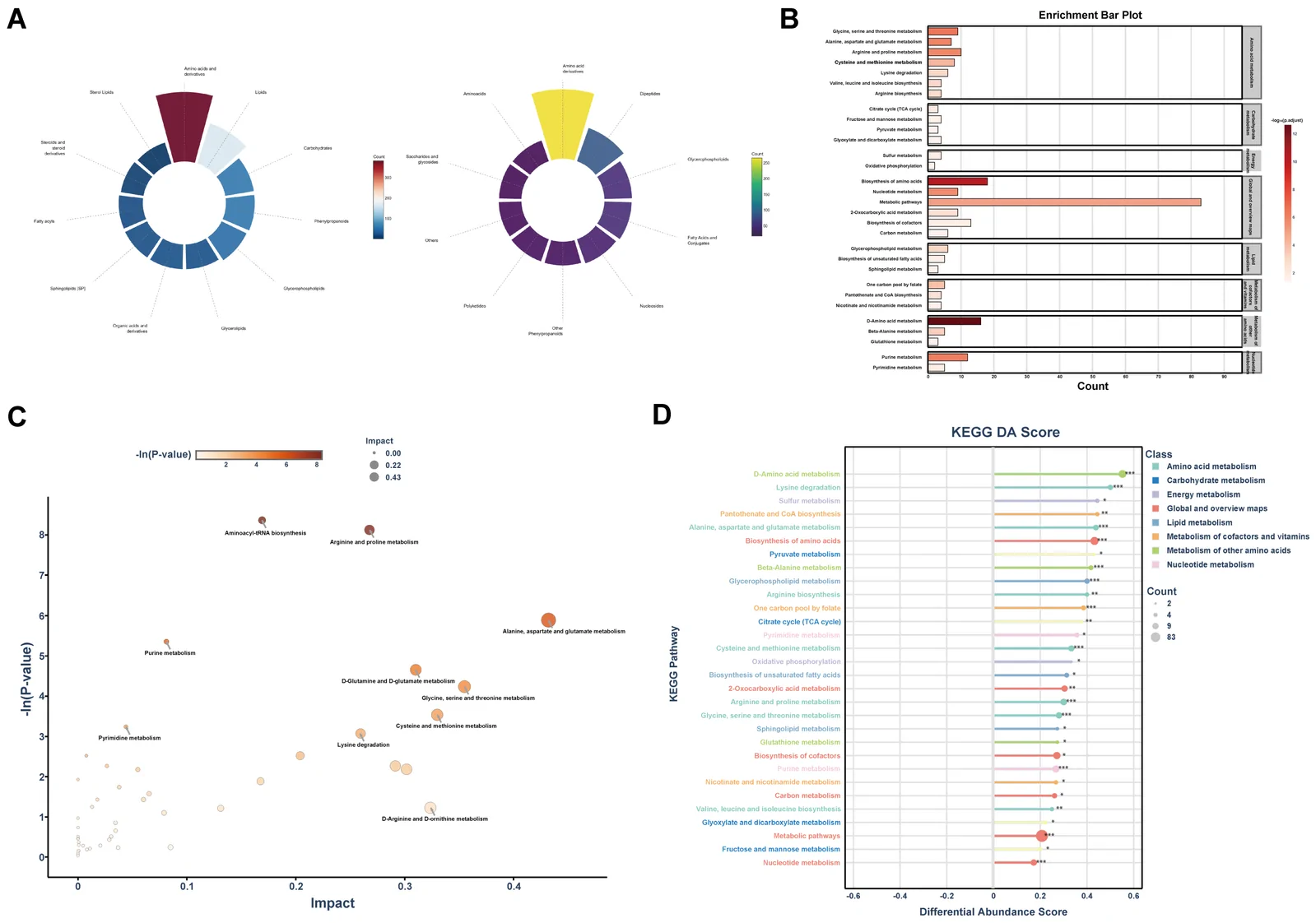
Metabolite classification and functional pathway analysis of DEMs. (**A**) Sunburst charts showing the classification (left) and subcategory distribution of identified DEMs (right). (**B**) KEGG pathway enrichment bar plot categorizing enriched pathways into primary metabolic modules. (**C**) Pathway topology analysis bubble plot (node size reflects Pathway Impact; color scale indicates *p* value). (**D**) Differential Abundance (DA) for the enriched KEGG pathways, illustrating the overall directional change in each pathway in LSCC tissues (* *p* < 0.05, ** *p* < 0.01, *** *p* < 0.001).

To further clarify the biochemical characteristics and metabolic perturbations associated with LSCC, chemical classification and pathway enrichment analyses were conducted using only the identified DEMs. Classification revealed that the DEMs spanned diverse structural classes, predominantly comprising amino acids and their derivatives, lipids and lipid-like molecules (e.g., glycerophospholipids, fatty acyls, and sterol lipids), organic acids, and carbohydrates (Figure 4A). KEGG pathway enrichment analysis demonstrated that these altered metabolites were broadly involved in key metabolic pathways, primarily centered on amino acid metabolism, carbohydrate metabolism, and nucleotide metabolism (Figure 4B). Pathway topology analysis further highlighted several top-impact metabolic pathways, including aminoacyl-tRNA biosynthesis, arginine and proline metabolism, alanine, aspartate, and glutamate metabolism, as well as purine metabolism (Figure 4C). Additionally, Differential Abundance (DA) score analysis of the enriched KEGG pathways indicated an overall upregulation trend across most altered metabolic pathways in LSCC tissues, particularly those governing amino acid turnover and energy biosynthesis (Figure 4D). Consistently, gene set enrichment analysis (GSEA) applied to these metabolic signatures further validated the directional activation of these enriched cascades, confirming a systematic enrichment toward globally upregulated metabolic states in LSCC tissues compared to adjacent normal counterparts (Figure S3). Collectively, these exploratory results indicate that a substantial proportion of the key metabolic alterations in LSCC specimens reflect increased amino acid utilization, nucleotide biosynthesis, and energy metabolism.

### 3.5. Integrated Analysis of Proteomics and Metabolomics

**Figure 5:**
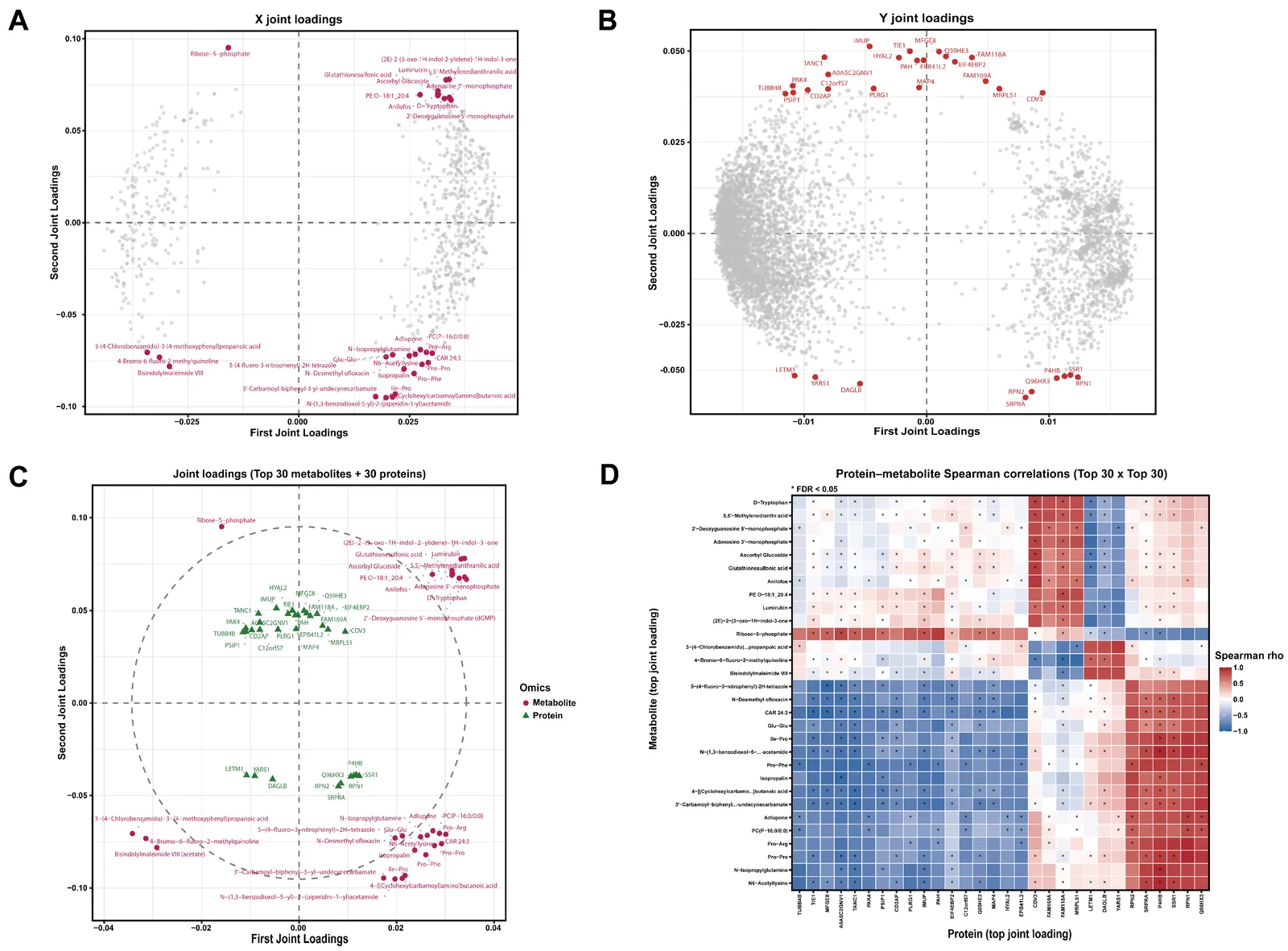
Integrated O2PLS and correlation analysis of proteomic and metabolomic data. (**A**) O2PLS joint loading plot for metabolomics (*X*-dataset), highlighting top contributing metabolites in magenta. (**B**) O2PLS joint loading plot for proteomics (*Y*-dataset), highlighting top contributing proteins in red. (**C**) Joint loading plot displaying the spatial distribution and co-clustering of the top 30 metabolites (magenta circles) and top 30 proteins (green triangles). (**D**) Spearman correlation heatmap showing pairwise associations between the top 30 metabolites and top 30 proteins derived from O2PLS loadings (* FDR < 0.05; color bar denotes Spearman correlation coefficient ρ).

To explore potential associations between the proteome and the metabolome, a bidirectional Orthogonal Partial Least Squares (O2PLS) integration model and Spearman correlation analysis were performed, and model robustness was evaluated by 1000-times permutation testing and cross-validation. O2PLS joint loadings from the metabolomic profile revealed key differential metabolites contributing significantly to the joint variance, including Ribose-5-phosphate, D-Tryptophan, Glutathionesulfonic acid, etc. (Figure 5A). Meanwhile, joint loadings from the proteomic profile highlighted pivotal proteins with high loading weights, such as TIE1, HYAL2, MFGE8, etc. (Figure 5B). The combined loading plot featuring the top 30 metabolites and top 30 proteins demonstrated strong spatial co-localization of key molecular features across different quadrants (Figure 5C). Specifically, metabolites in the upper-right quadrant (e.g., Adenosine 3′-monophosphate, D-Tryptophan, and 2′-Deoxyguanosine 5′-monophosphate) clustered closely with proteins including MRPL51, EIF4EBP2, MAP4, and FAM118A, indicating strong positive associations. Conversely, proteins localized in the lower-right quadrant, such as RPN1, RPN2, P4HB, and SSR1 (components involved in protein processing in the endoplasmic reticulum), co-clustered with a series of dipeptides, amino acids, and acylcarnitines (e.g., Pro-Phe, CAR 24:3). Spearman correlation analysis further described these integrated relationships (Figure 5D). A distinct bi-modular correlation structure was observed: a specific cluster of functional proteins (RPN1, RPN2, SSR1, P4HB, SRPRA, YARS1, DAGLB, LETM1) exhibited significant and pervasive positive correlations (*FDR < 0.05) with the lower-quadrant metabolites, while displaying strong negative correlations with upper-quadrant metabolites like Ribose-5-phosphate. In summary, these descriptive analyses suggest that central metabolic intermediates co-vary with protein quality control and endoplasmic reticulum machinery in LSCC tissues. Because these integrative analyses are based on only six paired samples, they are reported as hypothesis-generating observations: correlation between proteomic and metabolomic alterations does not demonstrate direct regulation, and these relationships require validation in larger, independent cohorts.

### 3.6. External Validation of Prioritized Candidates

**Figure 6:**
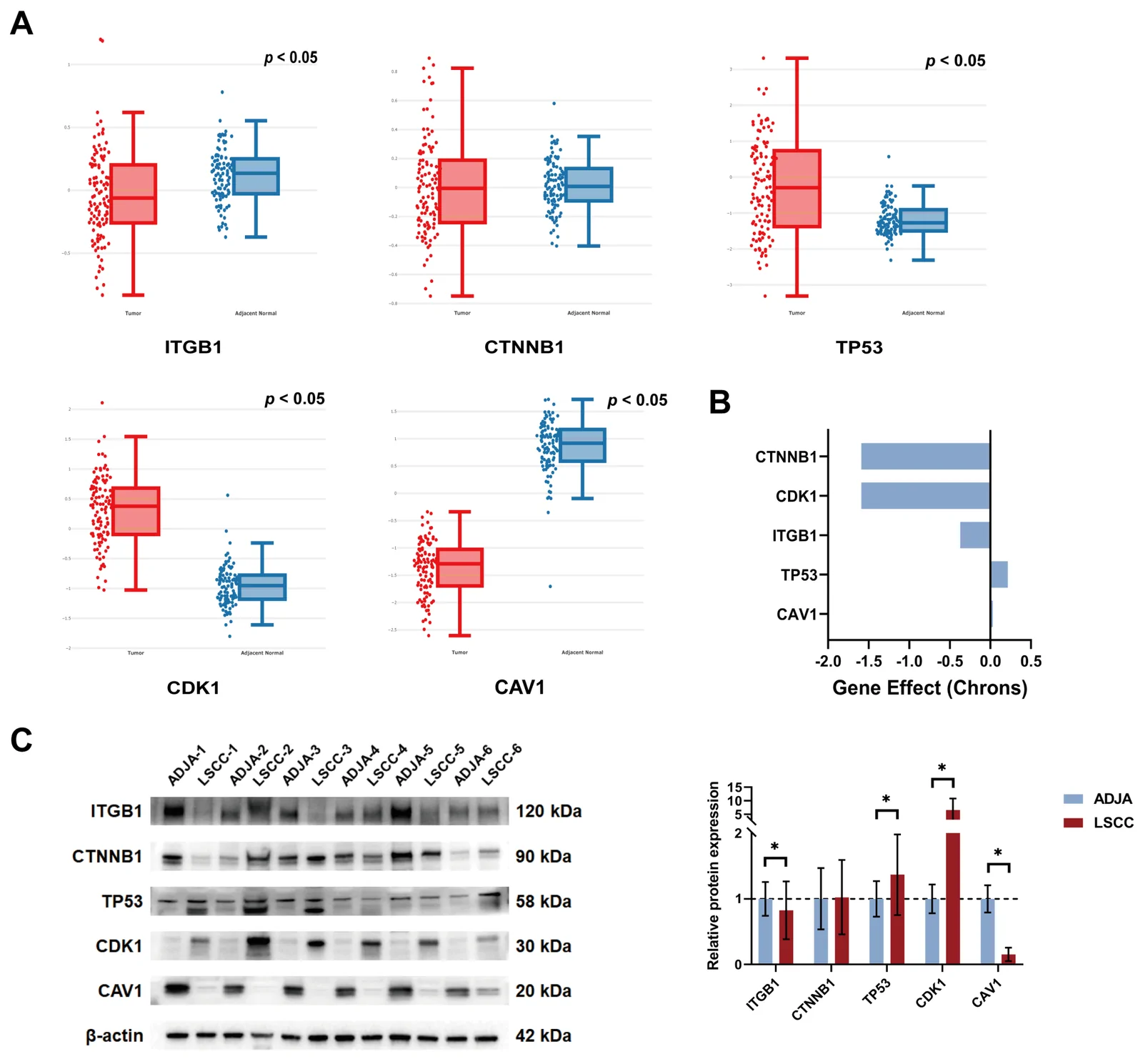
Cohort validation, functional essentiality profiling, and protein verification of prioritized hub candidates. (**A**) In silico external validation of protein expression levels for the five prioritized hub candidates using the CPTAC lung squamous cell carcinoma (LSCC) cohort (*n* = 108). (**B**) Gene dependency landscape of the prioritized candidates in the LSCC cell line (LK-2) retrieved from the Dependency Map (DepMap) portal. (**C**) Western blot verification of the hub proteins in matched clinical LSCC specimens and paired adjacent non-cancerous lung tissues (*n* = 6). * *p* < 0.05.

To place the exploratory findings in a broader context, we performed an in silico external validation using the publicly available proteogenomic data of the Clinical Proteomic Tumor Analysis Consortium (CPTAC) LSCC cohort (*n* = 108). Consistent with our DIA-MS profiling, key hub proteins exhibited distinct expression alterations: CDK1 and TP53 were significantly upregulated in LSCC tissues compared to matched non-cancerous adjacent tissues, whereas ITGB1 and CAV1 displayed marked downregulation (Figure 6A). Next, to assess the functional essentiality of these candidates for LSCC cell fitness (LK-2 cell line), we interrogated CRISPR-Cas9 loss-of-function screens from the DepMap resource. Chronos dependency profiling revealed that genetic depletion of CTNNB1, CDK1, and ITGB1 induced substantial fitness defects (Chronos scores < 0), with CTNNB1 and CDK1 acting as profound essentiality drivers (Chronos scores < −1.5). Finally, Western blot analysis of 5 hub genes in matched LSCC tissues (*n* = 6) confirmed the altered expression dynamics (Figure 6C and Figure S4). Densitometric quantification verified significant protein accumulation of TP53 and CDK1, along with significant repression of ITGB1 and CAV1 in LSCC relative to adjacent tissues (* *p* < 0.05). Collectively, these multi-tier validations reinforce the clinical fidelity and functional relevance of our prioritized multi-omics targets.

## 4. Discussion

In this pilot study, we integrated proteomic and non-targeted metabolomic data from lung squamous cell carcinoma (LSCC) tissues and paired adjacent non-cancerous tissues. Exploratory analyses identified 1312 differentially expressed proteins (DEPs) and 826 differentially expressed metabolites (DEMs) (|log_2_FC| ≥ 1 with adjusted *p* < 0.05 for proteins; VIP > 1 with *p* < 0.05 for metabolites). Joint analysis of the two datasets indicated that metabolic pathways were among the most significantly enriched features. These profiles offer preliminary molecular insights into the systemic dysregulation of LSCC, providing a functional foundation for future mechanistic studies in expanded cohorts.

Functional enrichment analysis of the DEPs identified in our cohort revealed a prominent enrichment of cell adhesion-related processes. These observations suggest an altered cell adhesion state in LSCC. Bonastre et al. observed that most LSCC cases histopathologically manifest keratinization and/or intercellular bridges, with molecular profiles characterized by high transcript levels of genes encoding keratins, as well as proteins associated with intercellular junctions and cell polarity [15]. Targeted disruption of cell adhesion-related protein expression has been shown to modulate LSCC progression [16]. Consistent with these functional insights, Kyoto Encyclopedia of Genes and Genomes (KEGG) pathway analysis demonstrated significant enrichment in cell adhesion signaling pathways, including Focal adhesion, IgSF CAM signaling, and Tight junction, further underscoring alterations in the intercellular junctional state. The dissemination of malignant cells from primary tumors to distant organs is inherently coupled with dramatic changes in their interactions with extracellular matrix components [17], while intercellular adhesion and cell migration represent critical processes during the early stages of cancer metastasis [18]. Notably, a study evaluating tumor fragmentation (TF) as a quantitative metric for LSCC invasiveness revealed that high-TF tumor cells upregulate focal adhesion processes, thereby augmenting LSCC aggressiveness [19]. Consequently, we hypothesize that the DEPs identified in this study may participate in the regulation of intercellular junctions and thereby affect the structural integrity and tissue architecture of LSCC. However, this hypothesis requires more functional validation.

Protein–protein interaction (PPI) analysis of the DEPs initially identified TP53, CTNNB1, and ITGB1 as the most highly connected nodes. TP53 is a key tumor suppressor gene encoding the multifunctional transcription factor p53, which mediates diverse anti-proliferative responses upon DNA damage—such as regulating cell cycle arrest, apoptosis, senescence, metabolic control, and DNA repair—to prevent and suppress aberrant cell growth [20]. TP53 mutations are detectable in approximately 51% of LSCC cases [21] and represent a hallmark genomic alteration associated with lung cancer risk in Asian populations [22]. CTNNB1 forms an integral component of the adherens junction complex, binding to the carboxyl-terminal domain of the cytoplasmic tail of E-cadherin. It acts as a central coordinator in cell adhesion and motility networks while regulating signaling pathways governing tumor cell adhesion and proliferation [23]. Functional studies have established that targeting CTNNB1 expression significantly impacts LSCC cell proliferation, migration, invasion, and tumor growth in vivo [24]. Integrins are widely acknowledged to govern a broad spectrum of cancer cell functions, including adhesion, migration, invasion, proliferation, and survival [25]. Among them, the extracellular domain of the β1 subunit interacts with a wide range of extracellular matrix proteins, playing a pivotal role in both primary tumorigenesis and metastatic dissemination across diverse malignancies [26]. Furthermore, Li et al. demonstrated that ITGB1 enhances radioresistance in human non-small cell lung cancer cells by orchestrating the DNA damage response and epithelial–mesenchymal transition [27]. To validate our findings, we integrated CPTAC cohort data (*n* = 108), DepMap CRISPR screens, and Western blotting. CPTAC and Western blot results consistently confirmed the upregulation of CDK1 and TP53 alongside the downregulation of ITGB1 and CAV1, highlighting aberrant cell cycle progression and cell–matrix dysregulation in LSCC. Notably, DepMap profiling identified CDK1 and CTNNB1 as critical essentiality drivers, establishing CDK1 as both overexpressed and indispensable for LSCC cell survival. Overall, these convergent validations reinforce the functional relevance and clinical fidelity of our prioritized candidates. However, these hub assignments are derived from a small discovery dataset and require validation in independent cohorts and functional models.

Unbiased non-targeted metabolomic profiling identified differentially abundant metabolites predominantly categorized as amino acids, lipids, and carbohydrates. KEGG enrichment analysis demonstrated significant enrichment of multiple amino acid biosynthetic and metabolic pathways, with a substantial proportion of their constituent metabolites showing increased abundance in LSCC samples after FDR correction. Integrating the magnitude of differential expression with pathway topology analysis suggested that alanine, aspartate, and glutamate metabolism were particularly prominent. Metabolic reprogramming to sustain continuous cell growth and proliferation is recognized as an emerging hallmark of cancer [28], with many solid tumors displaying alterations such as enhanced glutaminolysis [29]. Kim et al. demonstrated that elevated concentrations of metabolites including glutamate and phenylalanine correlate with LSCC aggressiveness [30]. Similarly, Rocha et al. observed that glutamate and alanine levels were significantly elevated and positively correlated in LSCC relative to lung adenocarcinoma [31]. Collectively, these findings suggest the pivotal roles of these three amino acid classes in LSCC pathogenesis. Nevertheless, given the exploratory scope and limited cohort size of the present study, residual false positives cannot be fully excluded, and validation in larger independent cohorts remains imperative. As targeted absolute quantification of amino acid pools was not performed in this exploratory phase, rigorous targeted metabolomic assays are required in future studies to establish precise concentration changes.

In our O2PLS two-way orthogonal partial least squares analysis, amino acid metabolism co-clustered with and exhibited strong positive correlations with proteins involved in protein processing in the endoplasmic reticulum (ER)—including SSR1, P4HB, and SRPRA. This associative pattern suggests a functional link between ER proteostasis and amino acid metabolism. Crucially, given that the O2PLS model and pairwise Spearman correlations were derived from a pilot cohort, these findings are strictly interpreted as exploratory, hypothesis-generating observations, as covariance does not establish direct regulatory causality. Previous studies have described a reciprocal and homeostatic interplay between ER homeostasis and amino acid dynamics. Horzum et al. demonstrated that blocking ER protein export triggers the accumulation of misfolded proteins, which compensatory activates ER-associated degradation (ERAD) and consequently elevates cytosolic free amino acid pools [32]. Meanwhile, Zhang et al. revealed that glycine functions as a signaling metabolite that modulates ER calcium homeostasis and attenuates ER stress via the GLRA1-calmodulin signaling axis [33]. It should be noted, however, that the integrated proteomic and metabolomic analyses in the current study rely on bioinformatic correlations and cannot establish causality; they therefore require experimental verification through functional assays in larger, independent cohorts.

A primary limitation of this study is its modest sample size. Due to the considerable practical and technical challenges in obtaining strictly matched, high-quality paired tumor and adjacent non-cancerous fresh tissues with complete clinical annotations, this work was framed as a pilot, hypothesis-generating discovery cohort. Consequently, statistical power remains limited, and residual false positives cannot be fully ruled out. Several further limitations should be acknowledged. First, the cohort consisted predominantly of male patients (5/6) with early-stage disease (IA/IB), so the findings cannot be extrapolated to female patients or to advanced-stage disease. Second, although the paired design and paired statistical models control for fixed individual-level covariates such as age, sex, and smoking history, they do not account for additional clinical covariates such as tumor purity, histologic subtype, or treatment history.

In summary, this exploratory study provides a characterization of the proteomic and metabolomic landscapes in LSCC tissues, unveiling preliminary molecular signatures across multiple proteins, metabolites, and functional pathways. Specifically, our integrative analyses highlight concerted dysregulation in cell adhesion states and amino acid metabolism, which may be linked through ER protein processing machinery. While exploratory in nature, these integrated omics findings offer valuable hypothesis-generating insights into LSCC pathogenesis and establish a foundational framework for prioritizing candidate biomarkers and therapeutic avenues for future large-scale validation.

## 5. Conclusions

This pilot omics study provides an exploratory, hypothesis-generating framework for the molecular landscape of LSCC. By implementing strict FDR-controlled differential profiling across matched tumor and adjacent non-cancerous tissues, we uncovered coordinated perturbations spanning cell adhesion architecture, amino acid turnover, and ER protein-processing machinery. While these associative networks represent a pilot-scale discovery, they offer meaningful insights into the multi-omics architecture of LSCC that warrant further expansion. Validating these integrative signatures through targeted quantification, orthogonal protein assays, and functional models in larger, multi-center cohorts will be essential to translate these exploratory insights into clinically actionable biomarkers.

## Figures and Tables

**Table 1:** Clinical characteristics of patients included in proteomic and metabolomic analyses.

Variables		All Participants (*n* = 6)
**Age**		62 ± 6.73
**Sex**	Male	5
	Female	1
**Stage**	pT1bN0M0, IA	2
	pT2aN0M0, IB	3
	pT1bN0M0, IA2	1
**Smoking status**	Nonsmoker	4
	Current smoker	2

## Data Availability

The mass spectrometry proteomics data and untargeted metabolomics raw data have been deposited to the National Genomics Data Center (Project number: PRJCA075931). All other data supporting the findings of this study are available from the corresponding authors upon reasonable request.

## Supplementary Materials

The following supporting information can be downloaded at: https://www.mdpi.com/article/10.3390/biomedicines14102389/s1, Figure S1: Histopathological H&E staining of patient tumor; Figure S2: O2PLS permutation diagnostics; Figure S3: Gene set enrichment analysis (GSEA); Figure S4: Raw Western blot images.

## Abbreviations

The following abbreviations are used in this manuscript:

LSCC	Lung squamous cell carcinoma
MSI	Metabolomics standards initiative
H&E	Hematoxylin and eosin
CPTAC	Clinical proteomic tumor analysis consortium
DepMap	Dependency map
ER	Endoplasmic reticulum
NSCLC	Non-small cell lung cancer
DIA	Data-independent acquisition
BCA	Bicinchoninic acid
DEPs	Differentially expressed proteins
PPI	Protein–Protein Interaction
MCC	Maximal clique centrality
OPLS-DA	Orthogonal partial least squares-discriminant analysis
HILIC	Hydrophilic interaction liquid chromatography
RPLC	Reversed-phase liquid chromatography
DEMs	Differentially expressed metabolites
O2PLS	Two-way orthogonal partial least squares
FDR	False discovery rate
GO	Gene ontology
BP	Biological process
CC	Cellular component
MF	Molecular function
KEGG	Kyoto encyclopedia of genes and genomes
ADJA	Adjacent non-tumor tissue
PCA	Principal component analysis
TP53	Tumor protein 53
ITGB1	Integrin subunit beta 1
CTNNB1	Catenin beta 1
CAV1	Caveolin 1
CDK1	Cyclin-Dependent Kinase 1
DA	Differential abundance
