# Supplementary material for: Proteomic and Metabolomic Analyses of Lung Squamous Cell Carcinoma
Source: Biomedicines. 2026 Oct 19;14(10):2389. doi: 10.3390/biomedicines14102389 (PMC13702009; doi:10.3390/biomedicines14102389)

Figure S1 Histopathological H&E staining of patient tumor

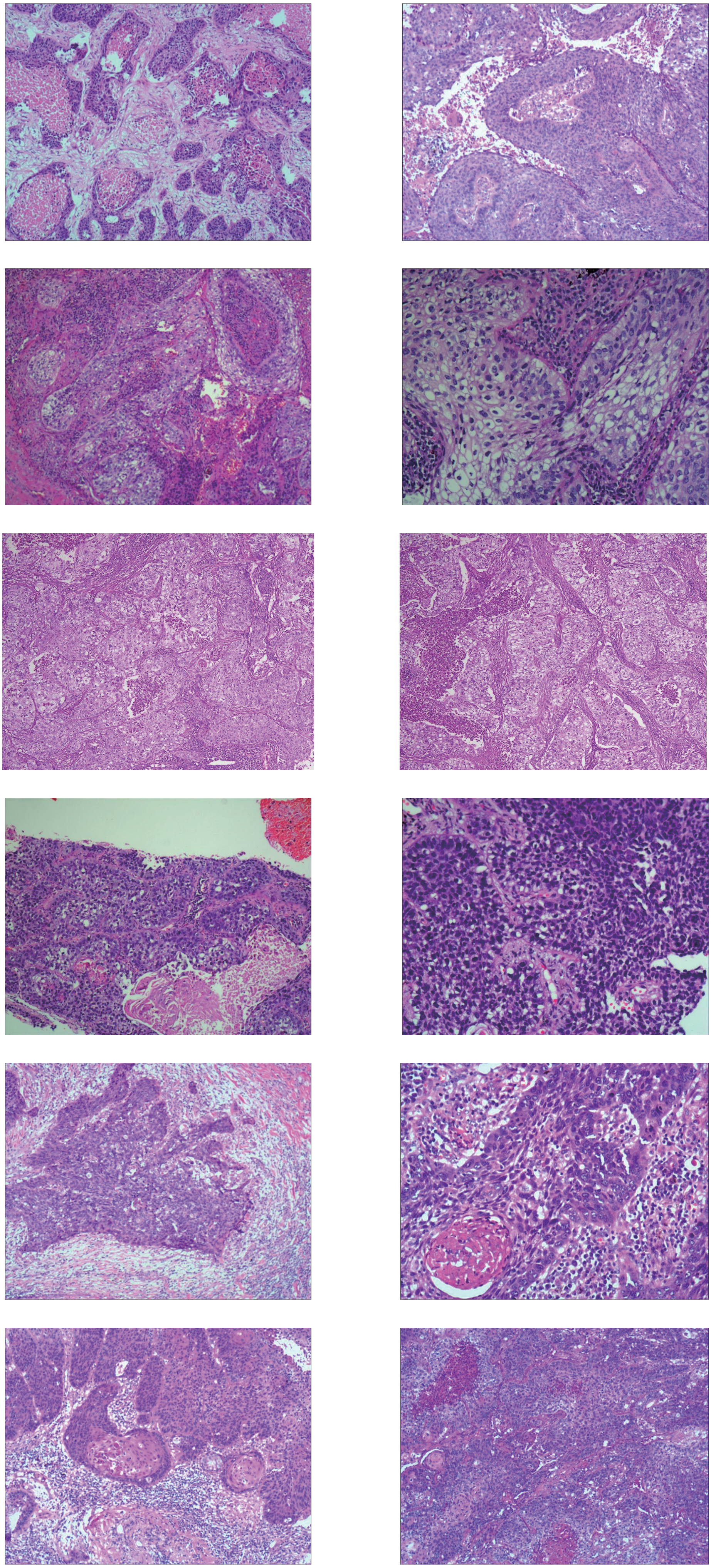

Figure S2 O2PLS permutation diagnostics

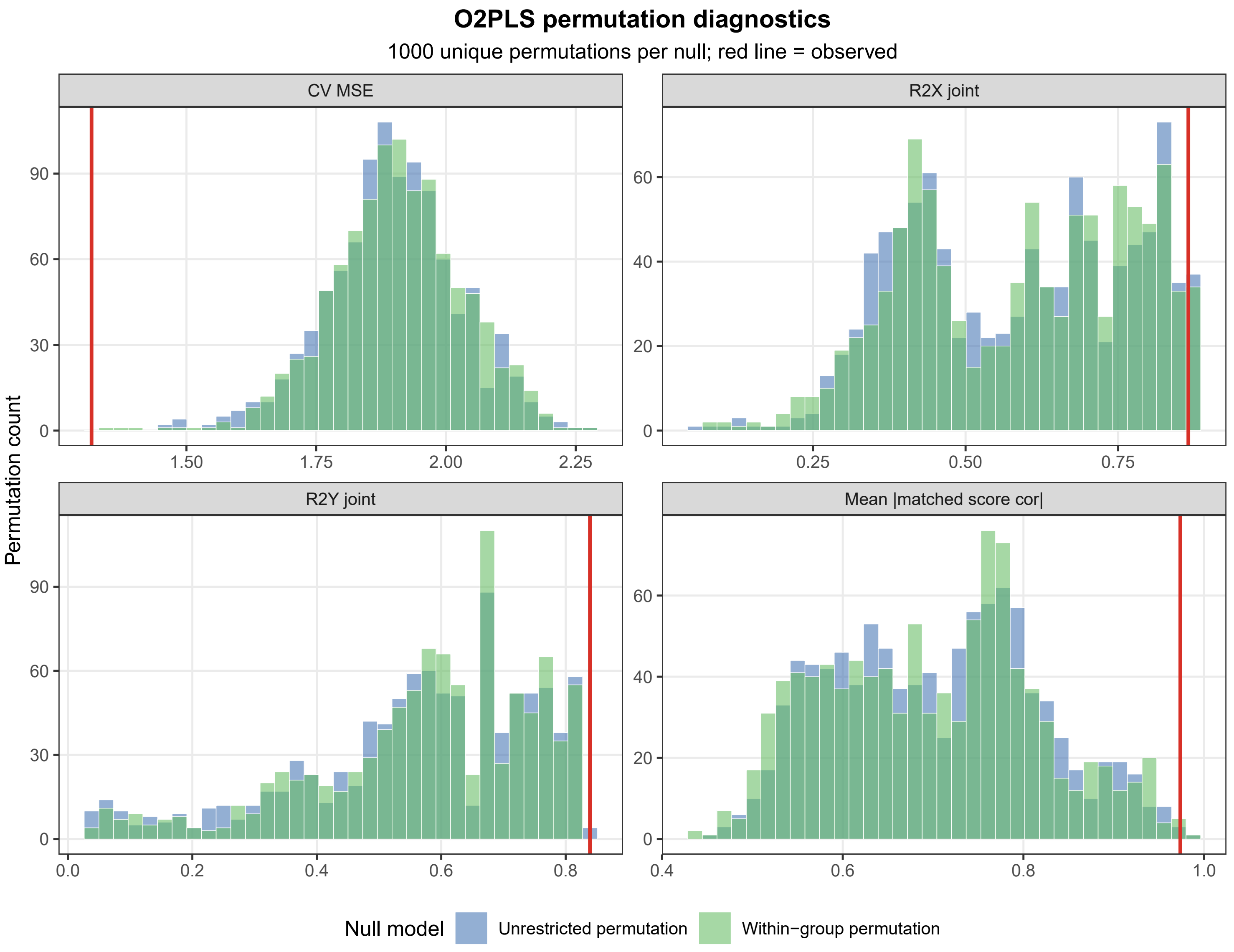

Figure S3 Gene set enrichment analysis (GSEA)

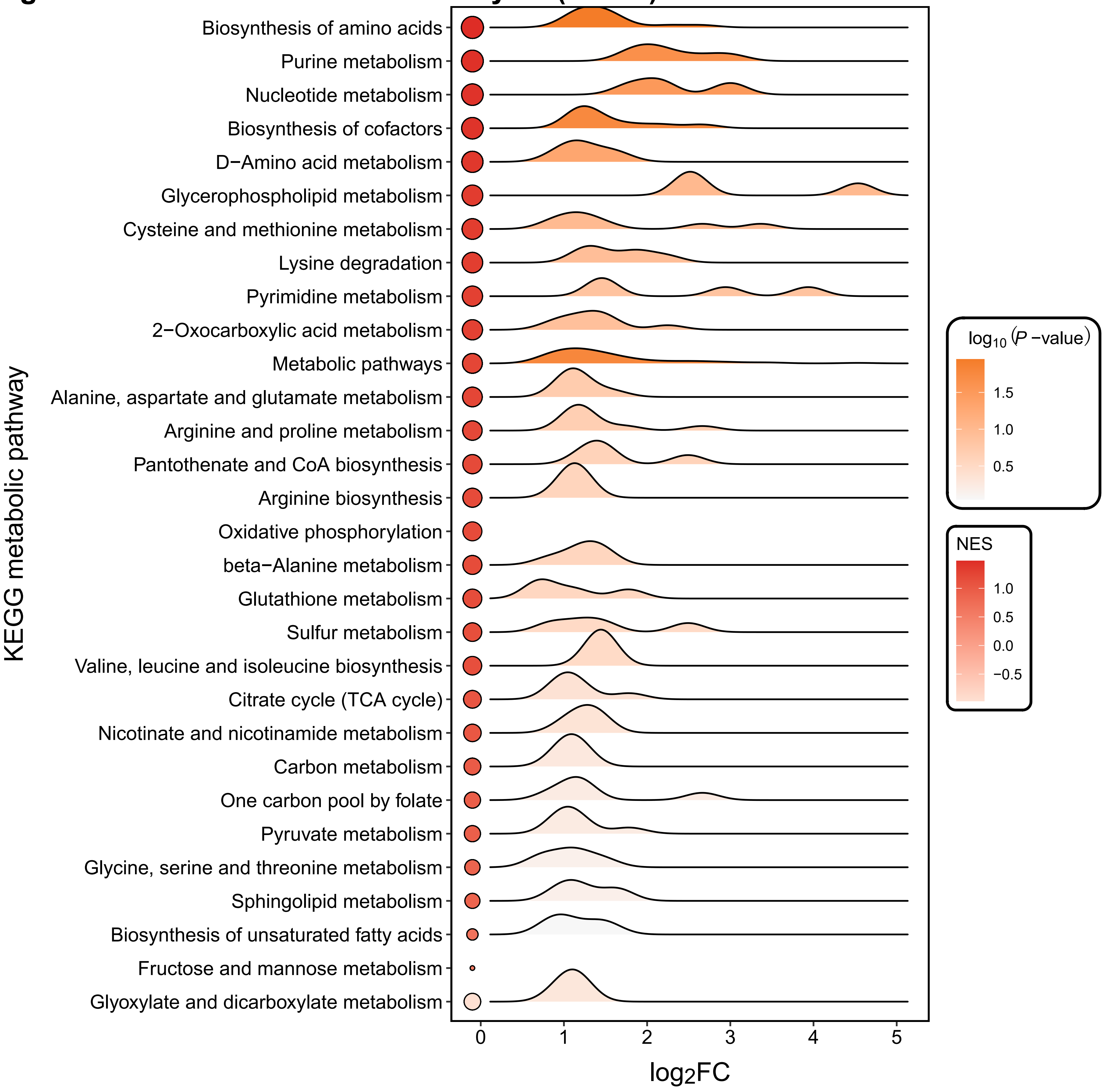

Figure S4 Raw western blot images

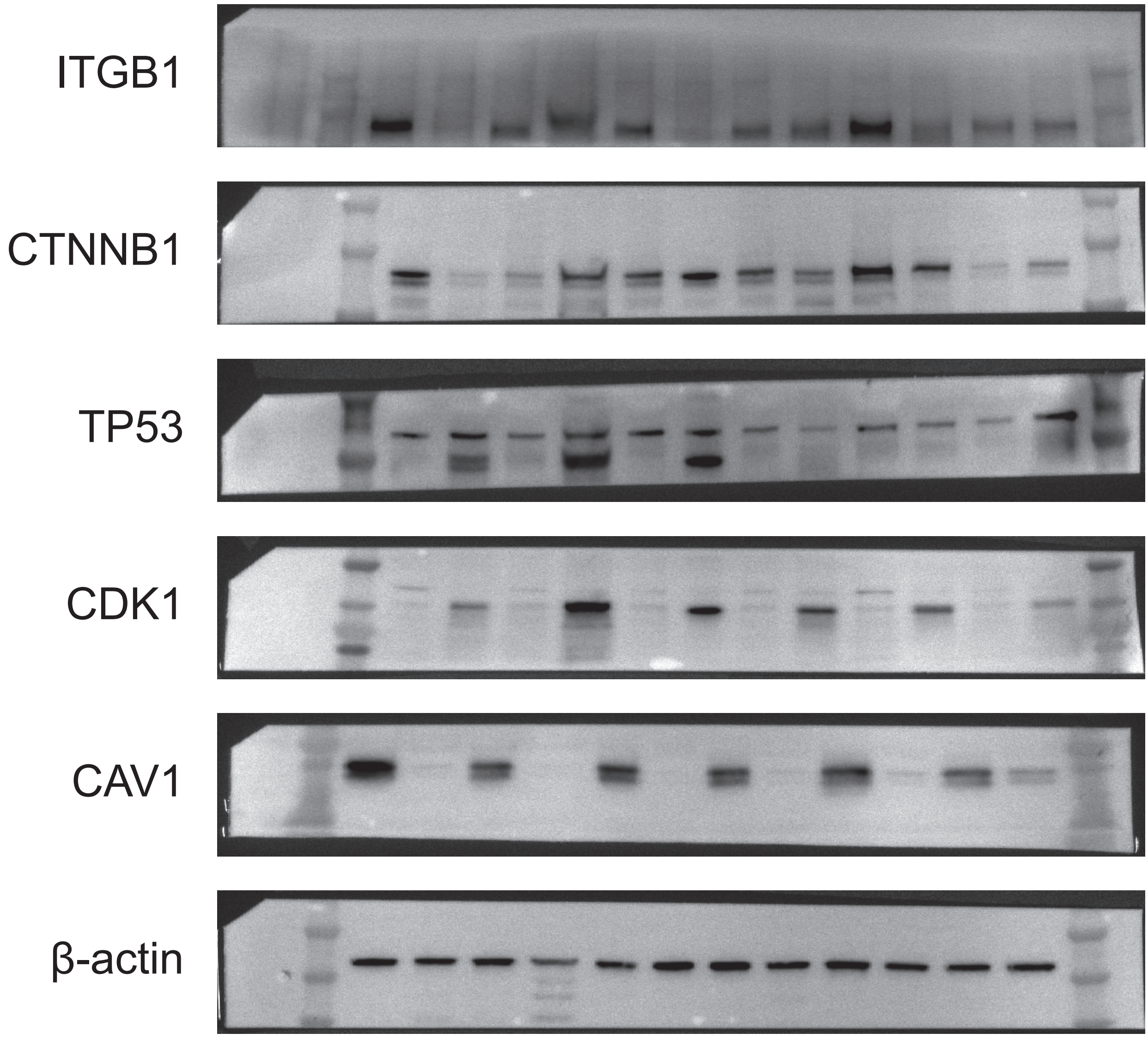

Supplement: Supplementary file 1 [file biomedicines-14-02389-s001.zip › biomedicines-4573645-Supplementary.pdf]
